# Long-Term Chemical-Only Fertilization Induces a Diversity Decline and Deep Selection on the Soil Bacteria

**DOI:** 10.1128/mSystems.00337-20

**Published:** 2020-07-14

**Authors:** Qicheng Xu, Ning Ling, Huan Chen, Yinghua Duan, Shuang Wang, Qirong Shen, Philippe Vandenkoornhuyse

**Affiliations:** a Jiangsu Provincial Key Lab for Solid Organic Waste Utilization, Jiangsu Collaborative Innovation Center for Solid Organic Waste Resource Utilization, Nanjing Agricultural University, Nanjing, Jiangsu, China; b Université de Rennes 1, CNRS, UMR 6553 EcoBio, Campus Beaulieu, Rennes, France; c Crop Research Institute, Anhui Academy of Agricultural Science, Hefei, China; d Institute of Agricultural Resources and Regional Planning, Chinese Academy of Agricultural Sciences, Beijing, China; e Heilongjiang Academy of Agricultural Sciences, Harbin, China; University of Illinois at Urbana-Champaign

**Keywords:** deterministic processes, fertilization, generalist, specialist, stochastic processes

## Abstract

Fertilization regimes are known to drive succession of the soil microbial community, whereas the assembly rules involved remain elusive. Moreover, the ecological roles of microbial “generalists” and “specialists” in soils with contrasting fertilization regimes have not been characterized. We explored how long-term fertilization regimes (i.e., treatments conducted for at least 30 years) impact the soil bacteria by modifying species richness, diversity, bacterial assembly, and niche breadth. Compared with long-term organic fertilizer input, the soils having undergone chemical-only fertilization contained smaller amounts of carbon resources and had a more acidic environment. This strong environmental constraint lowered the soil bacterial reservoir and resulted in a detectable ecoevolutionary transformation, with both a higher proportion of specialists and a stronger signature of deterministic processes. Overall, this study provided a new comprehensive understanding of the assembly rules of bacterial generalists and specialists under long-term fertilization regimes. This study also highlighted that chemical-only fertilization, a ubiquitous agricultural practice of current conventional agriculture, induced a strong and similar environmental force that transformed the soil microbiota from 28°N to 46°N included in this study.

**IMPORTANCE** Chemical-only fertilization is ubiquitous in contemporary conventional agriculture despite the fact that sustainability of this agricultural practice is increasingly being questioned because of the current observed soil degradation. We explored how long-term chemical-only versus organic-only fertilizations impacted the soil microbiota reservoir in terms of both diversity and induced assembly processes. The results showed that long-term chemical-only fertilization resulted in deep selection pressure on the soil microbial community reservoir, with both a higher proportion of specialists and a stronger signature of deterministic processes. The soil microbiota has clearly changed as a consequence of the fertilization regime. The diagnoses of the functional consequences of these ecoevolutionary changes in relation to agricultural practices are key to imagining agriculture in the time ahead and especially regarding future efforts for the conservation, restoration, and management of the soil microbiota reservoir which is key to the fertility of the ecosystem.

## Abstract (precis)

Chemical-only fertilization is ubiquitous in contemporary conventional agriculture despite the fact that sustainability of this agricultural practice is increasingly being questioned because of the current observed soil degradation. We explored how long-term chemical-only versus organic-only fertilizations impacted the soil microbiota reservoir in terms of both diversity and induced assembly processes. The results showed that long-term chemical-only fertilization resulted in deep selection pressure on the soil microbial community reservoir, with both a higher proportion of specialists and a stronger signature of deterministic processes. The soil microbiota has clearly changed as a consequence of the fertilization regime. The diagnoses of the functional consequences of these ecoevolutionary changes in relation to agricultural practices are key to imagining agriculture in the time ahead and especially regarding future efforts for the conservation, restoration, and management of the soil microbiota reservoir which is key to the fertility of the ecosystem.

## INTRODUCTION

Global food demand is increasing, and the premier challenge of our time is and will be to sustainably feed a growing human population (1). By 2050, global crop production may need to double (1). It is well-known that current conventional agriculture causes major threats to the environment, including a decline in biodiversity and related ecosystem services, water pollution, and soil degradation (e.g., reference 2), which jeopardize global food production. Ecologically intensive and sustainable agriculture is needed. In efforts to reach this goal, one neglected compartment is the soil biota (3), which plays a key role in the soil fertility ecosystem service (4).

Ecological diagnoses of the consequences of agricultural practices are fundamental for deciphering which practices could be best for maintaining the soil fertility ecosystem service. Within this framework, an increasing number of published studies have focused on the consequences of different farming practices (i.e., conventional versus organic farming) on “soil quality” (i.e., soil physical properties, chemical properties, and biological properties) (for a review, see reference 5). However, little is known about the effects of the practices on the ecoevolutionary ecology of soil organisms. Only a limited number of existing long-term field experiments allow assessment of the effect of particular farming constraints.

Among these farming constraints, it has been shown that fertilization regimes can result in distinct soil physical properties (e.g., aggregate composition and bulk density) and chemical properties (e.g., nutrient contents, pH, and electrical conductivity). The resulting changes in microhabitat are likely responsible for changes in soil microbial traits (6), including biomass, activity, and community structure and functioning (7, 8). However, the ecological processes driving the microbial community assembly in soils exposed to contrasting fertilization regimes remain rather unclear and overlooked.

Stochastic and deterministic processes are thought to simultaneously explain microbial community assembly (9). Disentangling these two key ecological processes enables measurement of the importance of environmental filtering (i.e., deterministic processes) relative to probabilistic dispersal, birth-death events, and ecological drift (i.e., stochastic processes) (10). In addition, generalist and specialist species statuses (i.e., niche breadth) are also known drivers of community composition in macroorganisms’ ecology, because within the framework of deterministic processes, the specialist-generalist paradigm predicts specialists to be more abundant due to a local advantage over generalists (11). Microorganism species that thrive in a wide variety of environmental conditions (i.e., generalists) have different assembly processes and different impacts on the dynamics of microbial community structure compared to species with narrower environmental distributions (i.e., specialists) (12, 13). We thus hypothesized that (i) fertilization regimes would result in changes in microbial community composition and (ii) these modifications would be more under the control of deterministic processes in the chemical-only fertilization regime because of strong selection pressure related to carbon depletion in these soils. We also tested the hypothesis (iii) of the existence of a higher proportion of specialist microorganisms in the chemical-only fertilization treatment and reciprocally more generalists in the organic treatment because of the availability of more-diverse C sources in the organic fertilization regime. Thus, (iv) compared to generalists, specialists would be more controlled by deterministic processes of assembly due to their higher dependence on particular environmental constraints.

To test these hypotheses, fields with long-term fertilization (i.e., more than 30 years), spanning approximately 28°N to 46°N across the major grain-producing areas in China, were selected to draw broad conclusions related to the consequences of the fertilization regime on bacterial communities. We used the null-model-based framework (14) to address the relative importance of stochastic and deterministic processes in the assemblies of bacterial specialists and generalists.

## RESULTS

### Comparison of soil properties and bacterial communities under different fertilization regimes.

Compared with chemical-only fertilization (CF) treatment, the organic fertilization treatments, including organic-only fertilization (OF) treatment and the combination of chemical and organic fertilization (COF), led to higher soil pH, soil organic carbon (SOC), total nitrogen (TN), and total phosphorus (TP) (see Table S1 in the supplemental material).

10.1128/mSystems.00337-20.6 TABLE S1 α-Diversity of bacterial communities and soil properties under different fertilization regimes. Download Table S1, DOCX file, 0.01 MB. Copyright © 2020 Xu et al. 2020 Xu et al. This content is distributed under the terms of the Creative Commons Attribution 4.0 International license.

In total, 2,501,984 high-quality sequences were obtained and clustered into 35,499 operational taxonomic units (OTUs) at a 97% sequence similarity level. After rarefying to 35,299 sequences per sample, 1,694,352 sequences belonging to 34,356 OTUs were retained. The sample-size-based rarefaction curve indicated that the subsample data set was reliable for further analysis (see Fig. S1 in the supplemental material).

10.1128/mSystems.00337-20.1 FIG S1 Rarefaction curves of sequencing data in different treatments. Download FIG S1, TIF file, 1.4 MB. Copyright © 2020 Xu et al. 2020 Xu et al. This content is distributed under the terms of the Creative Commons Attribution 4.0 International license.

In agreement with our working hypothesis, the bacterial communities in the chemical-only fertilization treatment had the lowest α-diversity, including the Shannon index and richness, compared with both organic-only treatment and the control (Table S1). Both field sites and fertilization regimes significantly contributed to the bacterial community differentiation (Fig. S2a). At the phylum level, *Proteobacteria* and *Acidobacteria* dominated whatever the treatments, accounting for 29 to 40% and 18 to 27% of the total sequences, respectively. Compared with other treatments, the control treatment had a more than 8% decrease in *Proteobacteria*. The organic-amended treatments (i.e., OF and COF) had a significantly higher relative abundance of *Bacteroidetes*, while the non-organic-amended treatments (i.e., control and CF) had a higher relative abundance of *Actinobacteria* and *Chloroflexi* (Table S2).

10.1128/mSystems.00337-20.2 FIG S2 Comparison of the entire bacterial communities (a), both the specialist and generalist communities (b), the pure generalist communities (c), and the pure specialist communities (d) in four long-term-fertilization experimental fields using nonmetric multidimensional scaling based on Bray-Curtis distances. Download FIG S2, TIF file, 1.7 MB. Copyright © 2020 Xu et al. 2020 Xu et al. This content is distributed under the terms of the Creative Commons Attribution 4.0 International license.

10.1128/mSystems.00337-20.7 TABLE S2 Compositions of bacterial communities at the phylum level under different fertilization regimes. Download Table S2, DOCX file, 0.01 MB. Copyright © 2020 Xu et al. 2020 Xu et al. This content is distributed under the terms of the Creative Commons Attribution 4.0 International license.

### Identification of the generalists and specialists.

**FIG 1:**
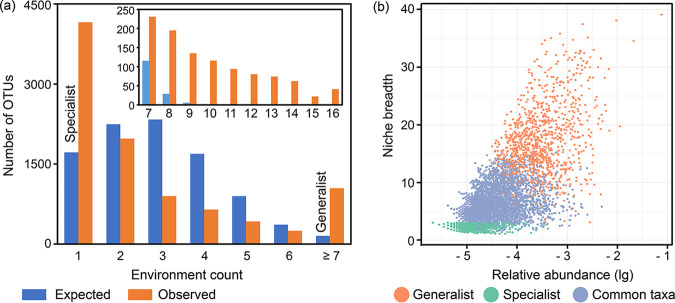
Classification of generalists and specialists. (a) Expected/observed OTU-environment distribution. The expected distribution was derived from 10,000 permutations. OTUs observed in one environment and six environments were defined as specialists and generalists, respectively. (b) Niche breadth of the specialists and generalists. Relative abundance is shown on a logarithmic (lg) scale on the *x* axis.

The generalist and specialist bacteria were identified from the OTU-environment association pattern. Based on comparisons of the observed distribution with the random distribution (see Materials and Methods for details), there were significant differences in the enrichment of OTUs belonging to one environment (i.e., specialists) or belonging to more than six environments (i.e., generalists) (Fig. 1a). A total of 1,050 OTUs (∼11%) accounting for ∼70% sequences (i.e., 1,008,616 sequences) were classified as generalists, while 4,157 OTUs (∼44%) from 44,369 sequences (∼3%) were classified as specialists; the remaining OTUs were considered common taxa. The richness of specialists was fourfold greater than that of generalists, but the relative abundance of the specialists was about 1/20 that of the generalists (Table 1). The distinction between the specialists and generalists was further validated by calculating the niche breadth of each OTU (Fig. 1b), which is a widely accepted way to divide specialists and generalists. As expected, generalists (orange dots in Fig. 1b) had a wider niche breadth than specialists (green dots in Fig. 1b). In soils of the chemical-only treatment, 1,018 OTUs (∼21%) from 226,416 sequences (∼62%) were classified as generalists, while 1,111 OTUs (∼23%) from 14,217 sequences (∼4%) were classified as specialists. Compared with chemical-only treatment, soils with organic amendment harbored more generalists (23 to 24% OTUs with 76 to 77% sequences) and fewer specialists (2% OTUs with 18 to 20% sequences) (Table 1).

**TABLE 1:** The number of OTUs and sequences of generalists, specialists, and common taxa in soils with long-term different fertilization regimes

Category	Parameter	No. of OTUs or sequences (%) in:				
		Meta community^*a*^	Control soil	CF soil	OF soil	COF soil
Generalist	No. of OTUs (%)	1,050 (11.16)	1,026 (20.04)	1,018 (21.41)	1,038 (22.83)	1,045 (24.31)
	No. of sequences (%)	1,008,616 (70.40)	238,164 (66.58)	226,416 (62.42)	275,763 (77.28)	268,273 (75.50)

Specialist	No. of OTUs (%)	4,157 (44.19)	1,345 (26.28)	1,111 (23.36)	909 (19.99)	792 (18.43)
	No. of sequences (%)	44,369 (3.10)	14,803 (4.14)	14,217 (3.92)	7,349 (2.06)	8,000 (2.25)

Common taxa	No. of OTUs (%)	4,200 (44.65)	2,748 (53.68)	2,626 (55.23)	2,600 (57.18)	2,461 (57.26)
	No. of sequences (%)	379,597 (26.50)	104,761 (29.28)	122,076 (33.66)	73,709 (20.66)	79,051 (22.25)

a Meta community referred to the entire community containing all the soil samples.

Field sites and fertilization regimes significantly impacted the community compositions of specialists and generalists (Fig. S2b). The subcommunities of generalists were clustered, but the subcommunities of specialists were dispersed (Fig. S2b). At the phylum level, generalists had a significantly higher abundance of *Proteobacteria*, *Acidobacteria,* and *Actinobacteria*. Specialists had a significantly higher abundance of *Planctomycetes*, *Bacteroidetes*, *Gemmatimonadetes*, *Chloroflexi*, “*Candidatus* Saccharibacteria,” and *Chlamydiae* (Table S3).

10.1128/mSystems.00337-20.8 TABLE S3 Compositions of specialist/generalist communities at the phylum level under different fertilization regimes. Download Table S3, DOCX file, 0.01 MB. Copyright © 2020 Xu et al. 2020 Xu et al. This content is distributed under the terms of the Creative Commons Attribution 4.0 International license.

**FIG 2:**
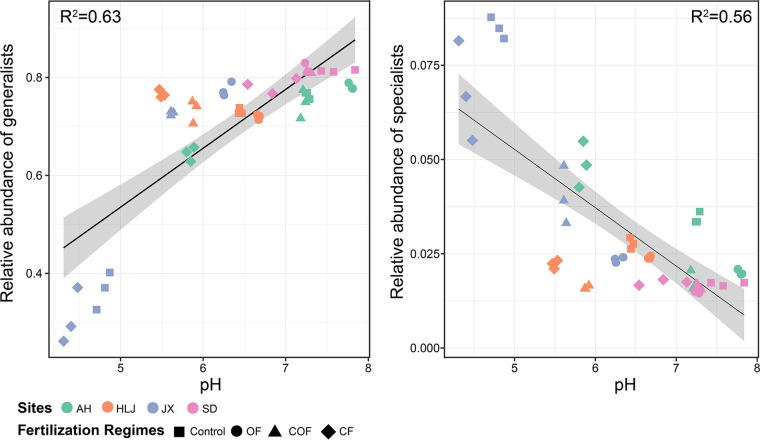
Linear regression analysis between the relative abundance of generalist/specialist and soil pH (*P* < 0.05 after correction). The sites and fertilization regimes are indicated by color and shape of the symbols, respectively.

The relative abundance of specialists/generalists was driven by the fertilization regime and related soil pH changes. The near-neutral soil pH leaded to higher abundance of generalists but lower abundance of specialists (Fig. 2). In addition to soil pH, the generalists increased with the increase in available phosphorus (AP), while the relative abundance of specialists decreased with increasing SOC, TN, NO_3_^−^, and C/N (Fig. S3). Overall, suitable environment (e.g., near-neutral soil pH and more available resources) increased the abundance of generalists but decreased the abundance of specialists.

10.1128/mSystems.00337-20.3 FIG S3 Linear regression analysis between the relative abundance of generalist/specialist and soil properties (*P* < 0.05 after correction). Red and green dots represented generalist and specialist groups, respectively. Download FIG S3, TIF file, 0.5 MB. Copyright © 2020 Xu et al. 2020 Xu et al. This content is distributed under the terms of the Creative Commons Attribution 4.0 International license.

### Bacterial assembly processes in different fertilization treatments.

**FIG 3:**
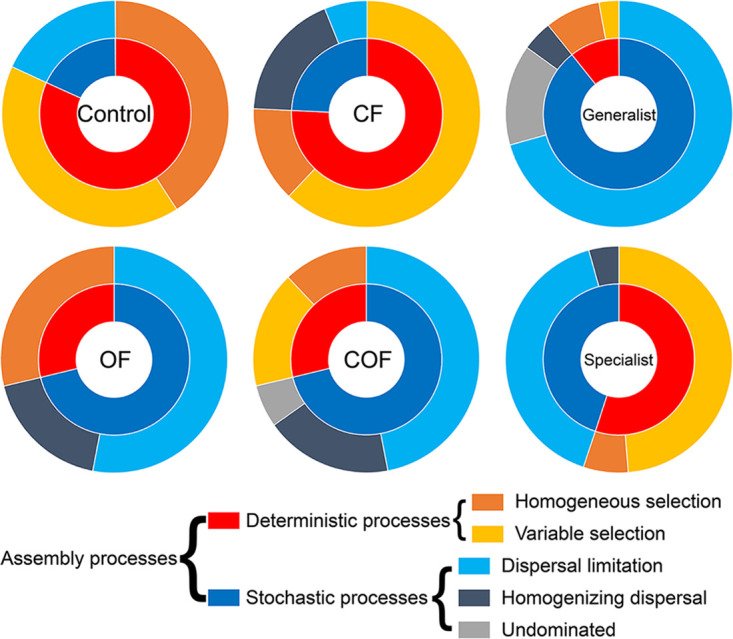
Assembly of the entire bacterial communities, specialists and generalists based on the null model. The inner circle represents the contribution of stochastic and deterministic processes to bacterial assembly. The outer circle represents the percentage of detailed ecological processes belonging to the stochastic or deterministic processes.

The null model was employed to explore the bacterial assembly processes in the entire bacterial communities, the generalist and specialist groups based on the phylogenetic signal analyses. Mantel correlograms were first used to test whether there was an association between phylogenetic relatedness and ecological similarity. They showed significant positive correlations (*P* < 0.05) across short phylogenetic distances in all groups (Fig. S4a). The fertilization regime altered the assembly processes of the bacterial community (Fig. 3 and Fig. S4). In agreement with our working hypothesis, the bacterial assembly in chemical-only soils was dominated by deterministic processes (∼76%), while in organic-amended soils (i.e., OF and COF), the assembly of the bacterial community was dominated by stochastic processes (∼71%) (Fig. 3). In addition, the assembly of the generalists was dominated by stochastic processes in all treatments (Fig. S4). In all treatments, the assembly of specialists was more influenced by deterministic processes than that of generalists (Fig. 3 and Fig. S4). Stochastic processes explained ∼32% of the assembly of specialists in chemical-only soils but explained 59 to 73% of that in organic-amended soils. Overall, the organic amendment dramatically increased the proportion of stochastic processes or limited the action of selection processes in the assembly of the entire bacterial community and the specialist groups.

10.1128/mSystems.00337-20.4 FIG S4 (a) Conservation of phylogenetic signals. Mantel correlation between the OTU niche distance and phylogenetic distance. A significant correlation (*P* < 0.05, solid squares) indicates a phylogenetic signal in species ecological niches. (b) Assembly of generalist/specialist in different fertilization treatments. The inner circle represents the contribution of stochastic and deterministic processes in bacterial assembly. The outer circle represents the percentage of detailed ecological processes belonging to the stochastic or deterministic processes. Download FIG S4, TIF file, 1.3 MB. Copyright © 2020 Xu et al. 2020 Xu et al. This content is distributed under the terms of the Creative Commons Attribution 4.0 International license.

**FIG 4:**
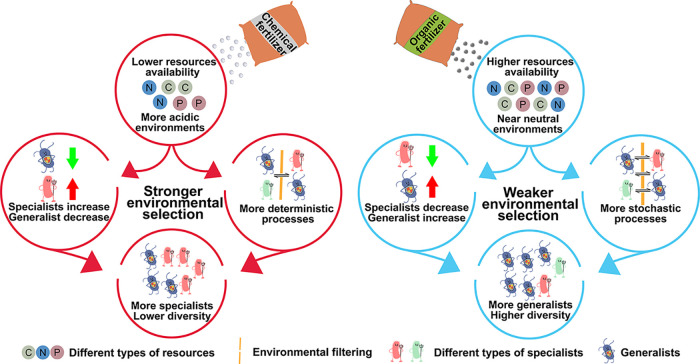
Impacts of long-term chemical- and organic-only fertilization on soil microbial communities. The regimes induced modifications to different soil parameters, including available nutrient resources and soil pH. As a consequence, the fertilization regimes induced different environmental stresses and assembly processes. Compared to organic-fertilized soil, the abundance of specialists was higher and the bacterial community assembly was more deterministic in the chemical-only fertilized soil.

Changes in the microbial community induced by fertilization regimes can be summarized as Fig. 4. Compared with organic fertilization, long-term chemical-only fertilization leaded to limited nutrient resources and acidic environment (Table S1), resulting in stronger environmental selection (Fig. 3). In this environment, specialists can be selected if they display improved competitive ability for resources and higher environmental adaptability (Fig. 2 and Fig. S3). As a consequence, there is a community with higher abundance of specialists and lower diversity in chemical-only fertilized soils compared with organic fertilized soils (Table 1). Long-term organic matter inputs to soils are associated with near-neutral soil pH, larger amounts of nutrient resources (Table S1), which leaded to a weaker environmental filtering and a higher proportion of generalists (Fig. 2 and Fig. S3), and finally resulted in a community with higher diversity (Table 1).

## DISCUSSION

### Long-term fertilization regimes induced changes in soil microbiota.

Long-term chemical-only fertilization is known to modify soil nutrients and soil structure, which will subsequently induce selection pressure on the soil microorganism reservoir (for a review, see reference 15). It has been shown, for instance, that long-term nitrogen fertilization decreases nitrification capacity and soil enzyme activities (16) and worse, suppresses N fixation ability of diazotrophs (6).

Herein, we found a clear decrease in bacterial community richness and diversity in long-term chemical-only fertilization treatment (see Table S1 in the supplemental material), which is in agreement with other studies (e.g., references 17 and 18). This soil microbiota reservoir degradation is likely a result of soil biogeochemical changes, including soil pH and organic carbon. Given the fact that microbial communities play central roles in supporting a variety of ecosystem services, including organic matter decomposition, nutrient cycling, and plant primary production, the observed decrease in the soil microbial reservoir is problematic. As recently emphasized, loss in soil microbial diversity negatively impacts the provision of services such as climate regulation, soil fertility, and food and fiber production (19). Even if agriculture practices have inherent negative effects on soil microbial diversity and functions, long-term chemical-only fertilization, as used in conventional agriculture in many countries around the world, is worse for the soil microbiota than organic fertilization regime.

### Ecological inferences to better understand the decline of the soil microbiota in chemical-only fertilized soils.

The ecological inferences on bacterial community assembly in relation to fertilization regimes remain overlooked and should be studied. Previous microbial ecological studies inferred bacterial community assembly using methods such as variation partitioning analysis (VPA) or a neutral model (for a review, see reference 9), but neither VPA nor neutral-theory-based model consider phylogenetic information. We employed a null-model-based framework incorporating taxonomic and phylogenetic metrics to determine the relative contributions of deterministic and stochastic processes in a reliable and comprehensive way (20). As expected from our working hypotheses, very different ecological processes occurred in relation to soil fertilization regimes (Fig. 3). In contrast to soil microbial community assembly in the organic-amended treatments, deterministic processes were the primary driver of the microbial reservoir in the chemical-only fertilization treatment. This is in line with a previous study showing that deterministic processes corresponded to low soil nutrient condition, while stochastic processes increased with higher nutrient (e.g., soil organic matter [SOM]) (21). The long-term chemical-only fertilization treatment has induced conditions for microbial carbon starvation and related ecoevolutionary processes, i.e., niche/habitat filtration acting on the global and local soil microbial reservoir (22). Reciprocally, in organic-amended soils, carbon and likely other compounds are not limiting microbial resources. This can directly explain both the observed higher microbial species richness and diversity, and a noteworthy dominant stochastic process of community assembly. In addition, organic fertilization can introduce many exogenous bacteria into the soil in a stochastic way, and this may contribute to the high diversity, especially on a long-term scale. The importance of deterministic processes in community assembly can be linked to the level of environmental stress acting on the soil microbial reservoir, i.e., the strength of the environmental filtering. The striking differentiation of the contribution of deterministic and stochastic processes to soil bacterial community assembly among soil fertilization treatments is also manifested in the characterization of the microbial fraction (specialist versus generalist) of the soil community reservoir.

### Generalists, specialists, and assembly processes of soil microbiota.

Within the soil microbial communities, generalists/specialists were determined by two different approaches (Fig. 1) to strengthen interpretations and conclusions. Generalists are characterized by their ability to colonize a wide range of habitats. They are thus expected to be less affected or to better buffer environmental filtering and selection processes. Furthermore, their high abundance observed herein (Fig. 1b) would allow better stochastic dispersion. The generalists were mainly represented by three abundant phyla (*Proteobacteria*, *Acidobacteria,* and *Actinobacteria*) (Table S3). Both *Proteobacteria* and *Actinobacteria* are often reported to be dominant and widely distributed in soil (23, 24). *Acidobacteria* are often considered to be slow growing with low nutrient requirements and are thus more resistant to nutrient limitation and can develop a viable population in a variety of habitats (25). Conversely, specialists are expected to have stricter conditions allowing growth, including specific requirements and/or dependencies on species interaction(s). The assembly of specialists is thus expected to be much more susceptible to modification by environmental changes and environmental filtering. In agreement with this theoretical understanding, deterministic processes dominated the community composition of specialists (Fig. 3).

As a result of this long-term fertilization regime, soil parameters have gradually changed over time, for instance the carbon stock in soils. Changes in the microbial community are interpreted to be driven by these parameters (i.e., environmental selection), while microbial dispersal from the global microbial pool was not strong enough to homogenize the microbial communities across treatments. Specialist microorganisms could be selected for their strong competitive ability in specific niches. Furthermore, it can be suggested that the microbial communities in soil fertilized by chemicals have a more limited access to carbon resources. In this case, it is possible that microorganisms in these communities have developed weapons to fight the other microorganisms for nutrient resources, including antibiotic resistance (26) and production. To explore this kind of ecoevolutionary expectation, future research will have to focus on the microbial functions supported by these soil microbiota communities.

### Conclusion.

This study not only expands our fundamental understanding of fertilization regime-driven assembly processes of bacterial generalists and specialists on a large scale but also establishes new perspectives on the conservation, restoration, and management efforts targeting degraded cultivated land in terms of the soil microbiota reservoir. The diagnostic made herein has to be enriched with complementary knowledge related to competition and synergies within communities, and it is necessary to better understand the consequences of these long-term soil amendments on the evolution of the soil microorganism reservoir and related function modifications.

## MATERIALS AND METHODS

### Field sites and sample collection.

Four long-term-fertilization field experiments conducted across the major grain-producing areas in China were chosen to test our working hypotheses, spanning approximately 28°N to 46°N (see Fig. S5 in the supplemental material). The four long-term-fertilization field experiments were located in (i) Heilongjiang Province (HLJ) (126.6°E, 45.7°N), (ii) Shandong Province (SD) (120.7°E, 36.9°N), (iii) Anhui Province (AH) (116.7°E, 33.6°N), and (iv) Jiangxi Province (JX) (116.4°E, 28.3°N) and were established in 1980, 1978, 1981, and 1986, respectively. Each long-term field experiment contained four different fertilization treatments: control, a chemical-only fertilization treatment (CF), an organic-only fertilization treatment (OF), and a combination of chemical and organic fertilization treatment (COF). Each treatment had three independent plots (replicates), and all plots were arranged in a randomized block design. In total, 48 samples (4 long-term experimental sites × 4 treatments × 3 replicates) from the top 0 to 20 cm of bulk soil were collected. Detailed information for HLJ, SD, AH, and JX regarding the amount of fertilization and chemical characteristics of the initial field soil was provided in previous publications (see Table S4 in the supplemental material) (27–30).

10.1128/mSystems.00337-20.5 FIG S5 Locations of four long-term fertilization experiment sites. Download FIG S5, TIF file, 0.9 MB. Copyright © 2020 Xu et al. 2020 Xu et al. This content is distributed under the terms of the Creative Commons Attribution 4.0 International license.

10.1128/mSystems.00337-20.9 TABLE S4 Detailed information for HLJ, SD, AH, and JX field sites regarding the amounts of fertilization and chemical characteristics of the initial field soil. Download Table S4, DOCX file, 0.02 MB. Copyright © 2020 Xu et al. 2020 Xu et al. This content is distributed under the terms of the Creative Commons Attribution 4.0 International license.

### Measurement of soil properties.

The soil pH was measured with a soil-to-water ratio of 2.5 using a pH microprobe. Soil organic carbon (SOC) and total nitrogen (TN) were measured by a vario Macro cube element analyzer. Soil total phosphorus (TP) and available phosphorus (AP) were extracted with HF-HClO_4_ and sodium bicarbonate, respectively, and finally determined via molybdenum-blue colorimetry. Available potassium (AK) was extracted with ammonium acetate and measured by flame photometry. NH_4_^+^ and NO_3_^−^ were extracted with a CaCl_2_ solution and determined using a continuous-flow stream autoanalyzer.

### DNA extraction, amplification, and sequencing.

The DNA was extracted with a DNeasy PowerSoil kit (Qiagen, Hilden, Germany) following the manufacturer’s instructions. The quantity of the extracted DNA was checked using a NanoDrop ND-1000 UV-visible (UV-Vis) spectrophotometer (NanoDrop Technologies, Wilmington, DE).

The primers 338F (5′-ACTCCTACGGGAGGCAGCAG-3′) and 806R (5′-GGACTACHVGGGTWTCTAAT-3′) were used for PCR amplification of the V3-4 region of the 16S rRNA gene for bacteria. The bacterial communities were characterized by high-throughput sequencing on an Illumina MiSeq instrument (Illumina, San Diego, CA, USA) at Sangon Biotechnology Co., Ltd. (Shanghai, China). The obtained sequence data were analyzed by USEARCH according to the UPARSE pipeline (31). Briefly, the forward and reverse reads were merged, and low-quality sequences (quality score < 20, length < 200 bp, or total expected errors > 0.5) were filtered. The remaining sequences were clustered into operational taxonomic units (OTUs) at a 97% identity threshold. The most abundant sequence of each OTU was considered the representative sequence and subsequently identified using the RDP Classifier. The singleton was removed to avoid possible biases accordingly (32).

### Definition of specialists and generalists.

The generalists and specialists were classified according to a recent study (33). Herein, each treatment was considered a unique environment, and we had a total of 16 environments (4 fertilization treatments × 4 experimental sites). The number of observed OTUs was calculated in each environment; then, 10,000 random permutations of the species-environment association map were performed (the number of OTUs in each environment was preserved) to obtain a random background distribution. The enrichment of the number of observed OTUs compared with the random distribution indicated that the OTUs were specialists/generalists.

Complementarily, the distinction between the generalists and specialists was validated using niche breadth with the formula below:

$$\text{niche breadth}=\frac{1}{\sum_{i=1}^{N}P_{\mathrm{ij}}^{2}}$$

where *P_ij_* is the relative abundance of OTU *j* in a given environment *i* (34). Generalists distributed across a wider range of environments have a higher niche breadth value than specialists.

### Bacterial assembly processes based on the null model.

The phylogenetic signal was assessed to determine whether there was an association between phylogenetic relatedness and ecological similarity. This step is essential for inferring ecological processes via the phylogenetic turnover between communities (10). A Mantel correlation between the OTU niche distance and phylogenetic distance with 999 randomizations was used to test the significance of the phylogenetic signals across the phylogenetic distances.

To determine the relative importance of deterministic and stochastic processes in bacterial assembly, a null model approach was used to analyze the phylogenetic diversity according to recent studies (35, 36). This framework is based on the phylogenetic turnover, which is the evolutionary distance separating OTUs in one community from OTUs in another community. After the calculation of abundance-weighted β-mean nearest taxon distance (βMNTD) (35), a distribution of βMNTD values was obtained by running the null model 999 times (βMNTD_null_). Then, the difference between βMNTD and the βMNTD_null_ distribution was referred to as the weighted beta nearest taxon index (βNTI). According to Graham and Stegen (36), values of βNTI that are less than −2 or greater than +2 are deemed significant in the sense that the observed βMNTD deviated significantly from the βMNTD_null_ distribution (36). According to Zhou and Ning (9), |βNTI| > 2 is a signature of nonrandom phylogenetic diversity (i.e., the control of deterministic processes). In this case, the sign of βNTI (i.e., βNTI > +2 and βNTI < −2) represents heterogeneous selection and homogeneous selection, respectively. When |βNTI| < 2, the corresponding community diversity results from a stochastic assembly process. In this case, the Bray-Curtis-based Raup-Crick (RCbray) index has to be considered. RCbray > 0.95 and RCbray < −0.95 represent dispersal limitation and homogenizing dispersal, respectively. |RCbray| < 0.95 represents the undominated scenario, which indicates that community turnover is dominated by multiple processes consisting of weak selection and dispersal, diversification, and/or drift (see reference 9 for details).

### Other statistical analyses.

The α-diversity (including the Shannon index and OTU richness) was calculated using mothur software. Variables were compared using a linear mixed model (*P* = 0.05). The rarefaction curves were conducted by the “iNEXT” package. Nonmetric multidimensional scaling (NMDS) based on Bray-Curtis distances and permutational multivariate analysis of variance (PERMANOVA) were conducted using the “vegan” and “ggplot2” packages in R (http://www.rproject.org/).

### Data availability.

The sequences obtained in this study are available from the NCBI Sequence Read Archive (https://www.ncbi.nlm.nih.gov/Traces/sra/) under accession number SRP193922.

## References

[1] Tilman D, Balzer C, Hill J, Befort BL 2011 Global food demand and the sustainable intensification of agriculture. Proc Natl Acad Sci U S A 108:20260–20264. doi:10.1073/pnas.1116437108. 22106295 PMC3250154

[2] Foley JA, Defries R, Asner GP, Barford C, Bonan G, Carpenter SR, Chapin FS, Coe MT, Daily GC, Gibbs HK, Helkowski JH, Holloway T, Howard EA, Kucharik CJ, Monfreda C, Patz JA, Prentice IC, Ramankutty N, Snyder PK 2005 Global consequences of land use. Science 309:570–574. doi:10.1126/science.1111772. 16040698

[3] Bender SF, Heijden MG 2015 Soil biota enhance agricultural sustainability by improving crop yield, nutrient uptake and reducing nitrogen leaching losses. J Appl Ecol 52:228–239. doi:10.1111/1365-2664.12351.

[4] Power AG 2010 Ecosystem services and agriculture: tradeoffs and synergies. Philos Trans R Soc Lond B Biol Sci 365:2959–2971. doi:10.1098/rstb.2010.0143. 20713396 PMC2935121

[5] Sheoran H, Kakar R, Kumar N 2019 Impact of organic and conventional farming practices on soil quality: a global review. Appl Ecol Environ Res 17:951–968. doi:10.15666/aeer/1701_951968.

[6] Fan F, Yu B, Wang B, George TS, Yin H, Xu D, Li D, Song A 2019 Microbial mechanisms of the contrast residue decomposition and priming effect in soils with different organic and chemical fertilization histories. Soil Biol Biochem 135:213–221. doi:10.1016/j.soilbio.2019.05.001.

[7] Sun R, Zhang X, Guo X, Wang D, Chu H 2015 Bacterial diversity in soils subjected to long-term chemical fertilization can be more stably maintained with the addition of livestock manure than wheat straw. Soil Biol Biochem 88:9–18. doi:10.1016/j.soilbio.2015.05.007.

[8] Xun W, Zhao J, Xue C, Zhang G, Ran W, Wang B, Shen Q, Zhang R 2016 Significant alteration of soil bacterial communities and organic carbon decomposition by different long‐term fertilization management conditions of extremely low‐productivity arable soil in South China. Environ Microbiol 18:1907–1917. doi:10.1111/1462-2920.13098. 26486414

[9] Zhou J, Ning D 2017 Stochastic community assembly: does it matter in microbial ecology? Microbiol Mol Biol Rev 81:e00002-17. doi:10.1128/MMBR.00002-17. 29021219 PMC5706748

[10] Stegen JC, Lin X, Konopka AE, Fredrickson JK 2012 Stochastic and deterministic assembly processes in subsurface microbial communities. ISME J 6:1653–1664. doi:10.1038/ismej.2012.22. 22456445 PMC3498916

[11] Mariadassou M, Pichon S, Ebert D 2015 Microbial ecosystems are dominated by specialist taxa. Ecol Lett 18:974–982. doi:10.1111/ele.12478. 26251267

[12] Pandit SN, Jurek K, Karl C 2009 Contrasts between habitat generalists and specialists: an empirical extension to the basic metacommunity framework. Ecology 90:2253–2262. doi:10.1890/08-0851.1. 19739387

[13] Székely AJ, Silke L 2014 The importance of species sorting differs between habitat generalists and specialists in bacterial communities. FEMS Microbiol Ecol 87:102–112. doi:10.1111/1574-6941.12195. 23991811

[14] Jia X, Dini-Andreote F, Salles JF 2018 Community assembly processes of the microbial rare biosphere. Trends Microbiol 26:738–747. doi:10.1016/j.tim.2018.02.011. 29550356

[15] Geisseler D, Scow KM 2014 Long-term effects of mineral fertilizers on soil microorganisms – a review. Soil Biol Biochem 75:54–63. doi:10.1016/j.soilbio.2014.03.023.

[16] Shen W, Lin X, Shi W, Min J, Gao N, Zhang H, Yin R, He X 2010 Higher rates of nitrogen fertilization decrease soil enzyme activities, microbial functional diversity and nitrification capacity in a Chinese polytunnel greenhouse vegetable land. Plant Soil 337:137–150. doi:10.1007/s11104-010-0511-2.

[17] Dai Z, Su W, Chen H, Barberán A, Zhao H, Yu M, Yu L, Brookes PC, Schadt CW, Chang SX, Xu J 2018 Long-term nitrogen fertilization decreases bacterial diversity and favors the growth of Actinobacteria and Proteobacteria in agro-ecosystems across the globe. Glob Chang Biol 24:3452–3461. doi:10.1111/gcb.14163. 29645398

[18] Wang C, Liu D, Bai E 2018 Decreasing soil microbial diversity is associated with decreasing microbial biomass under nitrogen addition. Soil Biol Biochem 120:126–133. doi:10.1016/j.soilbio.2018.02.003.

[19] Delgado-Baquerizo M, Maestre FT, Reich PB, Jeffries TC, Gaitan JJ, Encinar D, Berdugo M, Campbell CD, Singh BK 2016 Microbial diversity drives multifunctionality in terrestrial ecosystems. Nat Commun 7:10541. doi:10.1038/ncomms10541. 26817514 PMC4738359

[20] Stegen JC, Lin X, Fredrickson JK, Chen X, Kennedy DW, Murray CJ, Rockhold ML, Konopka A 2013 Quantifying community assembly processes and identifying features that impose them. ISME J 7:2069–2079. doi:10.1038/ismej.2013.93. 23739053 PMC3806266

[21] Feng Y, Chen R, Stegen JC, Guo Z, Zhang J, Li Z, Lin X 2018 Two key features influencing community assembly processes at regional scale: initial state and degree of change in environmental conditions. Mol Ecol 27:5238–5251. doi:10.1111/mec.14914. 30368967

[22] Thuiller W, Münkemüller T, Lavergne S, Mouillot D, Mouquet N, Schiffers K, Gravel D 2013 A road map for integrating eco‐evolutionary processes into biodiversity models. Ecol Lett 16:94–105. doi:10.1111/ele.12104. 23679011 PMC3790307

[23] Janssen PH 2006 Identifying the dominant soil bacterial taxa in libraries of 16S rRNA and 16S rRNA genes. Appl Environ Microbiol 72:1719–1728. doi:10.1128/AEM.72.3.1719-1728.2006. 16517615 PMC1393246

[24] Fierer N 2017 Embracing the unknown: disentangling the complexities of the soil microbiome. Nat Rev Microbiol 15:579–590. doi:10.1038/nrmicro.2017.87. 28824177

[25] Hartman WH, Richardson CJ, Rytas V, Bruland GL 2008 Environmental and anthropogenic controls over bacterial communities in wetland soils. Proc Natl Acad Sci U S A 105:17842–17847. doi:10.1073/pnas.0808254105. 19004771 PMC2584698

[26] Wang F, Xu M, Stedtfeld RD, Sheng H, Fan J, Liu M, Chai B, Soares de Carvalho T, Li H, Li Z, Hashsham SA, Tiedje JM 2018 Long-term effect of different fertilization and cropping systems on the soil antibiotic resistome. Environ Sci Technol 52:13037–13046. doi:10.1021/acs.est.8b04330. 30375866

[27] Zhong W, Gu T, Wang W, Zhang B, Lin X, Huang Q, Shen W 2010 The effects of mineral fertilizer and organic manure on soil microbial community and diversity. Plant Soil 326:511–522. doi:10.1007/s11104-009-9988-y.

[28] Song X, Liu S, Liu Q, Zhang W, Hu C 2014 Carbon sequestration in soil humic substances under long-term fertilization in a wheat-maize system from North China. J Integr Agric 13:562–569. doi:10.1016/S2095-3119(13)60713-3.

[29] Guo J, Ling N, Chen H, Zhu C, Kong Y, Wang M, Shen Q, Guo S 2017 Distinct drivers of activity, abundance, diversity and composition of ammonia-oxidizers: evidence from a long-term field experiment. Soil Biol Biochem 115:403–414. doi:10.1016/j.soilbio.2017.09.007.

[30] Wang Q, Jiang X, Guan D, Wei D, Zhao B, Ma M, Chen S, Li L, Cao F, Li J 2018 Long-term fertilization changes bacterial diversity and bacterial communities in the maize rhizosphere of Chinese Mollisols. Appl Soil Ecol 125:88–96. doi:10.1016/j.apsoil.2017.12.007.

[31] Edgar RC 2013 UPARSE: highly accurate OTU sequences from microbial amplicon reads. Nat Methods 10:996–998. doi:10.1038/nmeth.2604. 23955772

[32] Liu L, Yang J, Yu Z, Wilkinson DM 2015 The biogeography of abundant and rare bacterioplankton in the lakes and reservoirs of China. ISME J 9:2068–2077. doi:10.1038/ismej.2015.29. 25748371 PMC4542038

[33] Sriswasdi S, Yang C, Iwasaki W 2017 Generalist species drive microbial dispersion and evolution. Nat Commun 8:1162. doi:10.1038/s41467-017-01265-1. 29079803 PMC5660117

[34] Liao J, Cao X, Zhao L, Wang J, Gao Z, Wang MC, Huang Y 2016 The importance of neutral and niche processes for bacterial community assembly differs between habitat generalists and specialists. FEMS Microbiol Ecol 92:fiw174. doi:10.1093/femsec/fiw174. 27543321

[35] Stegen JC, Lin X, Fredrickson JK, Konopka AE 2015 Estimating and mapping ecological processes influencing microbial community assembly. Front Microbiol 6:370. doi:10.3389/fmicb.2015.00370. 25983725 PMC4416444

[36] Graham E, Stegen J 2017 Dispersal-based microbial community assembly decreases biogeochemical function. Processes 5:65. doi:10.3390/pr5040065.

