# Supplementary material for: Long-Term Chemical-Only Fertilization Induces a Diversity Decline and Deep Selection on the Soil Bacteria
Source: mSystems. 2020 Jul 14;5(4):e00337-20. doi: 10.1128/mSystems.00337-20 (PMC7363003; doi:10.1128/mSystems.00337-20)
Supplement: TABLE S1 [file mSystems.00337-20-st001.docx]

Table S1 The α-diversity of bacterial communities and soil properties under different fertilization regimes.

|  | Shannon | Richness | pH | NH_4_^+^-N (mg/kg) | NO_3_^-^-N (mg/kg) | AP (mg/kg) | AK (mg/kg) | SOC (g/kg) | TN (g/kg) | TP (g/kg) | C/N |
| --- | --- | --- | --- | --- | --- | --- | --- | --- | --- | --- | --- |
| Control | 6.77 a | 4434 a | 6.53 b | 1.57 b | 4.00 c | 5.27 c | 73.92 a | 9.53 b | 1.06 c | 0.42 b | 8.80 a |
| CF | 6.44 c | 3878 b | 5.64 c | 2.88 ab | 17.39 b | 38.78 bc | 118.42 a | 11.29 b | 1.31 b | 0.61 b | 8.62 a |
| OF | 6.72 ab | 4470 a | 6.99 a | 4.53 a | 24.25 a | 149.94 a | 112.67 a | 15.56 a | 1.72 a | 1.16 a | 9.05 a |
| COF | 6.56 bc | 4229 ab | 6.50 b | 3.34 ab | 22.04 ab | 110.82 ab | 125.33 b | 13.51 a | 1.56 a | 1.17 a | 8.72 a |

Means (*n* = 12) ± standard deviations within a column followed by dissimilar letters indicate significance (*P* ≤ 0.05) according to the mixed liner model. The “Fertilization regimes” (Control, CF, OF and COF) factor is fixed, and the “Sites” (AH, HLJ, SD and JX) factor is considered as random effect.
