# Supplementary figures and images for: Long-Term Chemical-Only Fertilization Induces a Diversity Decline and Deep Selection on the Soil Bacteria
Source: mSystems. 2020 Jul 14;5(4):e00337-20. doi: 10.1128/mSystems.00337-20 (PMC7363003; doi:10.1128/mSystems.00337-20)

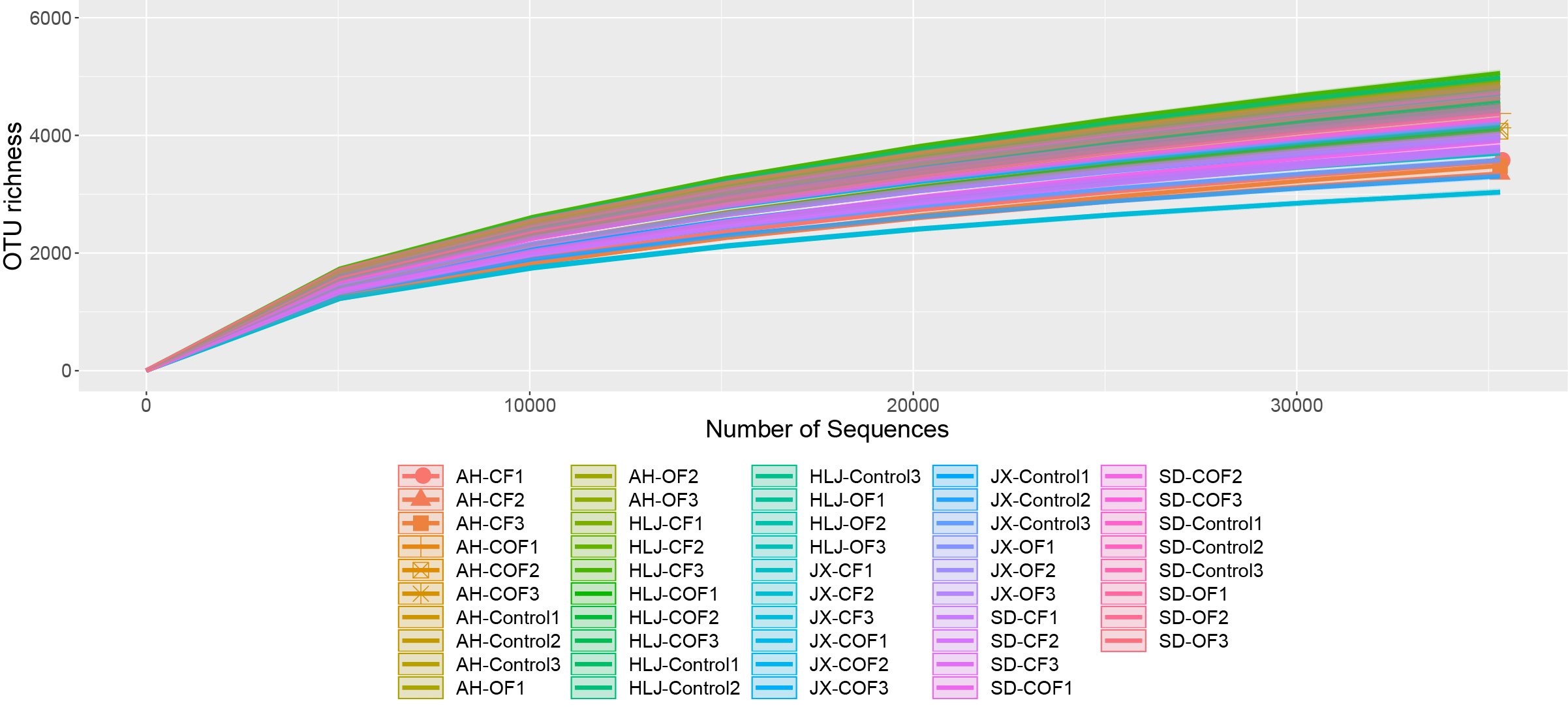

Supplement: FIG S1 [file mSystems.00337-20-sf001.tif]

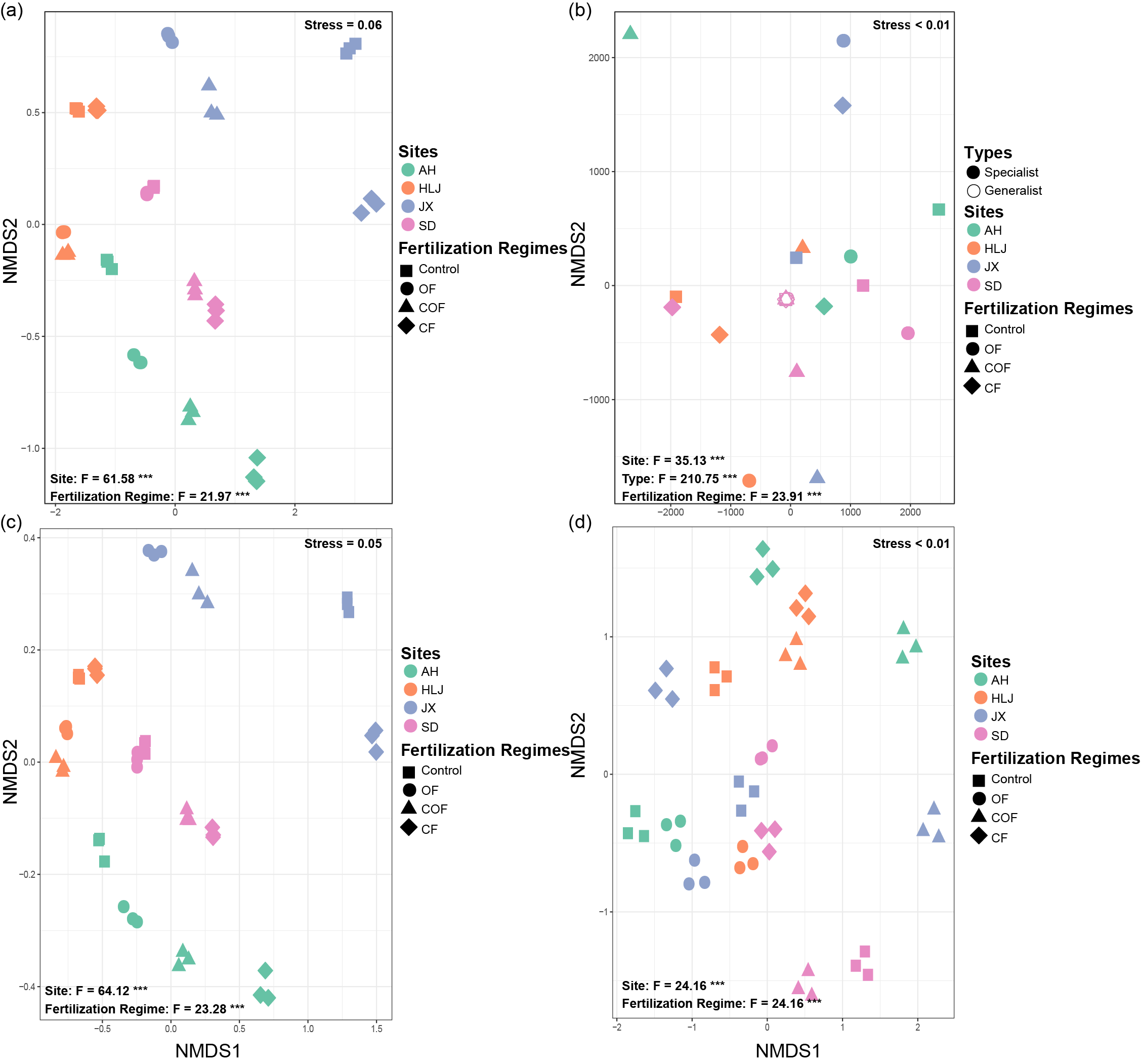

Supplement: FIG S2 [file mSystems.00337-20-sf002.tif]

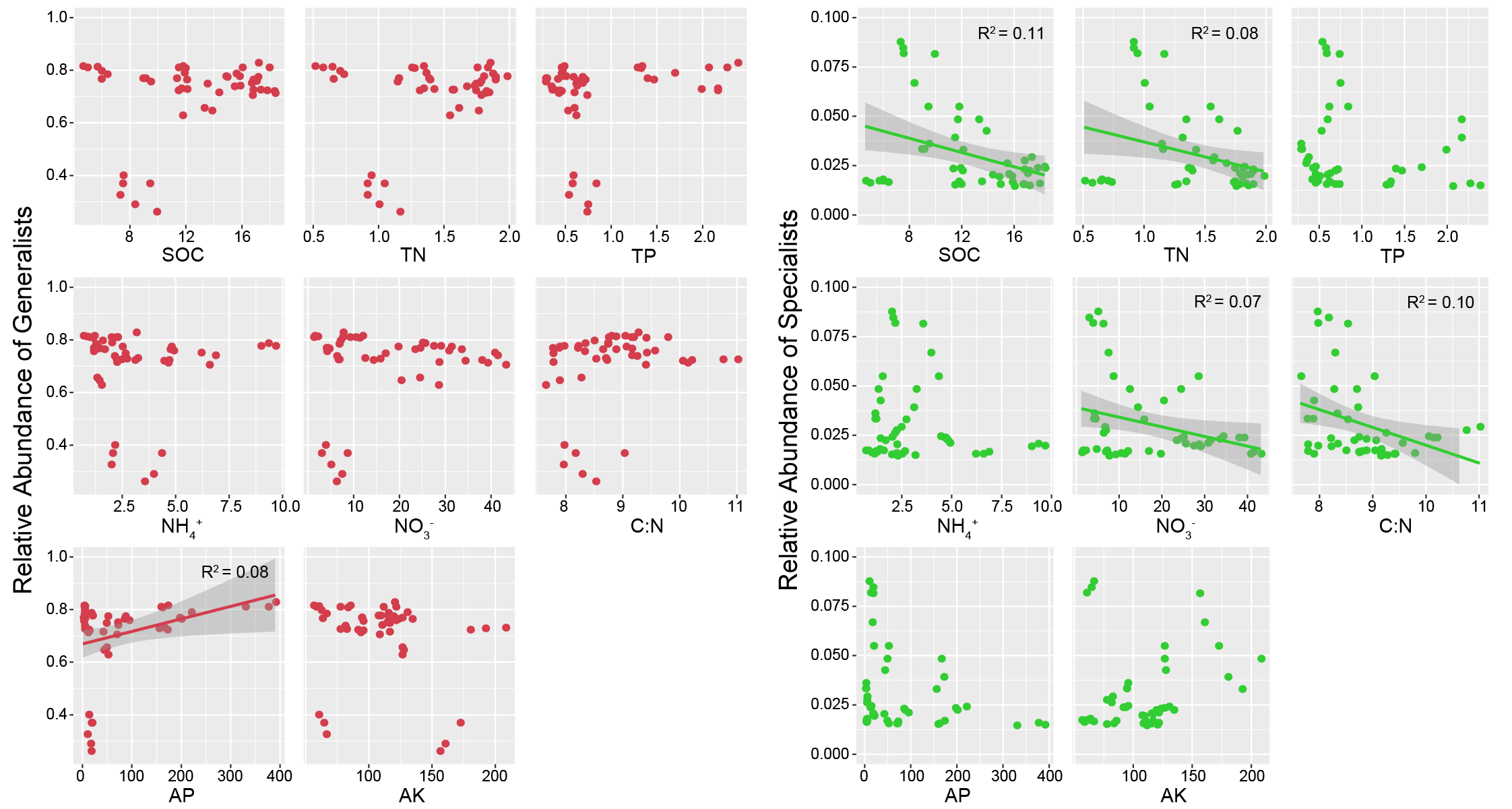

Supplement: FIG S3 [file mSystems.00337-20-sf003.tif]

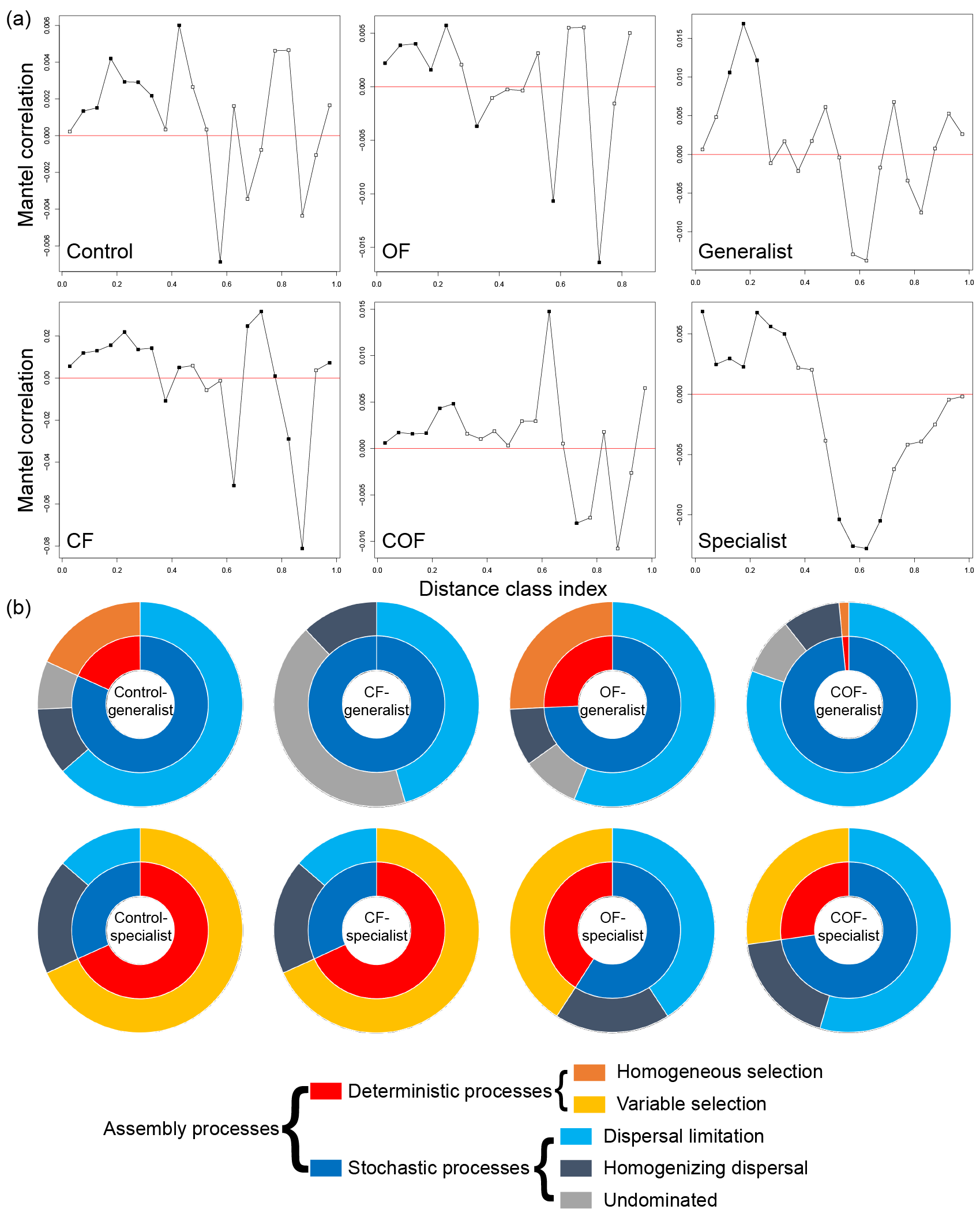

Supplement: FIG S4 [file mSystems.00337-20-sf004.tif]

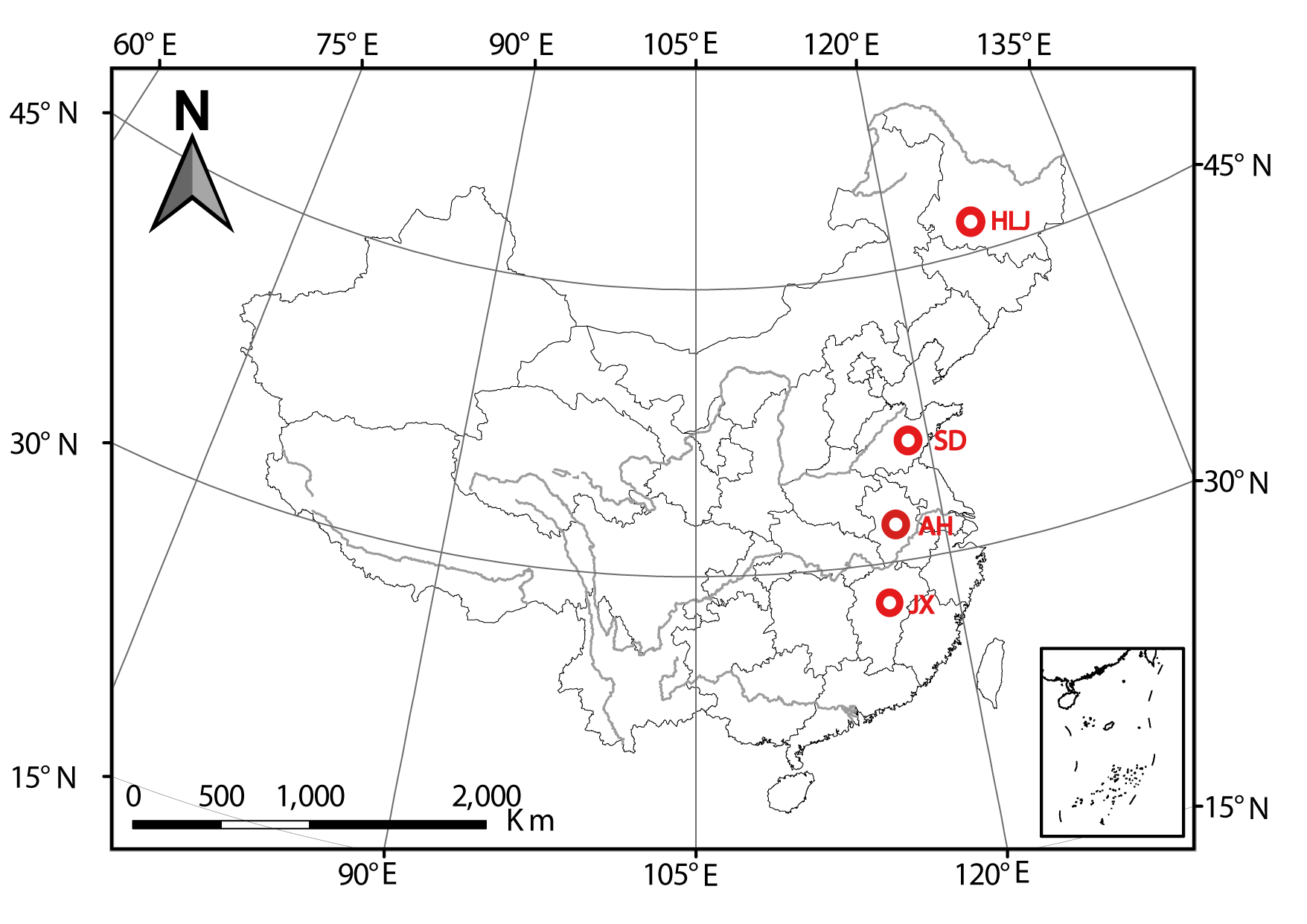

Supplement: FIG S5 [file mSystems.00337-20-sf005.tif]
