# Supplementary material for: Long-Term Chemical-Only Fertilization Induces a Diversity Decline and Deep Selection on the Soil Bacteria
Source: mSystems. 2020 Jul 14;5(4):e00337-20. doi: 10.1128/mSystems.00337-20 (PMC7363003; doi:10.1128/mSystems.00337-20)
Supplement: TABLE S2 [file mSystems.00337-20-st002.docx]

Table S2 The bacterial relative abundance at phylum level under different fertilization regimes.

|  | Control | CF | OF | COF |
| --- | --- | --- | --- | --- |
| *Proteobacteria* | 0.29 b | 0.37 a | 0.37 a | 0.40 a |
| *Acidobacteria* | 0.27 a | 0.20 bc | 0.24 ab | 0.18 c |
| *Actinobacteria* | 0.09 ab | 0.11 a | 0.07 b | 0.08 b |
| *Planctomycetes* | 0.05 a | 0.04 a | 0.05 a | 0.05 a |
| *Bacteroidetes* | 0.04 b | 0.05 b | 0.08 a | 0.07 a |
| *Chloroflexi* | 0.03 ab | 0.03 a | 0.01 b | 0.01 b |
| *Firmicutes* | 0.03 ab | 0.04 a | 0.02 b | 0.03 ab |
| *Verrucomicrobia* | 0.05 a | 0.03 b | 0.05 ab | 0.05 ab |
| *Gemmatimonadetes* | 0.04 a | 0.04 a | 0.04 a | 0.04 a |
| *WPS-1* | 0.01 a | 0.01 a | 0.02 a | 0.02 a |
| *Candidatus Saccharibacteria* | 0 c | 0.01 a | 0.01 bc | 0.01 ab |
| *WPS-2* | 0 ab | 0.01 a | 0 b | 0 b |
| *Nitrospirae* | 0.01 a | 0.01 a | 0.01 a | 0.01 a |
| Unclassified | 0.06 | 0.05 | 0.04 | 0.04 |
| Others | 0.01 | 0.01 | 0.01 | 0.01 |

Means (*n* = 12) ± standard deviations within a row followed by dissimilar letters indicate significance (*P* ≤ 0.05) according to the mixed liner model. The “Fertilization regimes” (Control, CF, OF and COF) factor is fixed, and the “Sites” (AH, HLJ, SD and JX) factor is considered as random effect.
