# Supplementary material for: Long-Term Chemical-Only Fertilization Induces a Diversity Decline and Deep Selection on the Soil Bacteria
Source: mSystems. 2020 Jul 14;5(4):e00337-20. doi: 10.1128/mSystems.00337-20 (PMC7363003; doi:10.1128/mSystems.00337-20)
Supplement: TABLE S3 [file mSystems.00337-20-st003.docx]

Table S3 The relative abundance of generalists/specialists at phylum level.

|  | Generalists | Specialists |
| --- | --- | --- |
| *Proteobacteria* | 0.42 a | 0.24 b |
| *Acidobacteria* | 0.24 a | 0.18 b |
| *Actinobacteria* | 0.10 a | 0.07 b |
| *Bacteroidetes* | 0.05 b | 0.09 a |
| *Verrucomicrobia* | 0.05 a | 0.04 a |
| *Gemmatimonadetes* | 0.03 b | 0.04 a |
| *Firmicutes* | 0.02 a | 0.03 a |
| *Planctomycetes* | 0.02 b | 0.09 a |
| *WPS-1* | 0.01 a | 0.01 a |
| *Chloroflexi* | 0.01 b | 0.04 a |
| *Candidatus Saccharibacteria* | 0.00 b | 0.04 a |
| *Chlamydiae* | 0 b | 0.01 a |
| Others | 0.01 | 0.02 |
| Unclassified | 0.03 | 0.10 |

Means (*n* = 48) ± standard deviations within a row followed by dissimilar letters indicate significance (*P* ≤ 0.05) according to the mixed liner model. The “Type” (generalist and specialist) factor is fixed. The “Fertilization regimes” (Control, CF, OF and COF) factor, and the “Sites” (AH, HLJ, SD and JX) factor are considered as random effects.
