# Supplementary material for: Long-Term Chemical-Only Fertilization Induces a Diversity Decline and Deep Selection on the Soil Bacteria
Source: mSystems. 2020 Jul 14;5(4):e00337-20. doi: 10.1128/mSystems.00337-20 (PMC7363003; doi:10.1128/mSystems.00337-20)
Supplement: TABLE S4 [file mSystems.00337-20-st004.docx]

Table S4 Detailed information for HLJ, SD, AH and JX regarding the amounts of fertilisation and chemical characteristics of the initial field soil.

| Site | Plot size  (m^2^) | pH | SOC  (g/kg) | Total N  (g/kg) | Total P  (g/kg) | Available P  (mg/kg) | Available K  (mg/kg) | Fertilisation  regimes | Inorganic fertilizer rate ^a^ [kg/(ha*year)] | | | Organic fertilizer rate ^b^ |
| --- | --- | --- | --- | --- | --- | --- | --- | --- | --- | --- | --- | --- |
|  |  |  |  |  |  |  |  |  | N | P_2_O_5_ | K_2_O | [kg/(ha*year)] |
| HLJ | 36 | 7.22 | 26.7 | 1.47 | 1.07 | 51 | 200 | Control | - | - | - | - |
|  |  |  |  |  |  |  |  | CF | 150 | 75 | 75 | - |
|  |  |  |  |  |  |  |  | OF | - | - | - | ~6,200 |
|  |  |  |  |  |  |  |  | COF | 150 | 75 | 75 | ~6,200 |
| SD | 33 | 6.8 | 2.38 | 0.5 | 0.46 | 15 | 38 | Control | - | - | - | - |
|  |  |  |  |  |  |  |  | CF | 276 | 90 | 135 | - |
|  |  |  |  |  |  |  |  | OF | - | - | - | ~30,000 |
|  |  |  |  |  |  |  |  | COF | 138 | - | - | ~30,000 |
| AH | 30 | 7.6 | 10.22 | 0.78 | 0.47 | 2.5 | 101 | Control | - | - | - | - |
|  |  |  |  |  |  |  |  | CF | 525 | 210 | 210 | - |
|  |  |  |  |  |  |  |  | OF | - | - | - | ~8750 |
|  |  |  |  |  |  |  |  | COF | 262.5 | 105 | 105 | ~4375 |
| JX | 22 | 5.36 | 8.59 | 1 | 0.57 | 12.75 | 91 | Control | - | - | - | - |
|  |  |  |  |  |  |  |  | CF | 120 | 60 | 120 | - |
|  |  |  |  |  |  |  |  | OF | - | - | - | ~30,000 |
|  |  |  |  |  |  |  |  | COF | 120 | 60 | 120 | ~30,000 |

^a^The mineral N, P, and K were applied as urea, superphosphate, and potassium chloride, respectively.

^b^The organic fertilizer is pig manure in SD and JX, is Horse manure in HLJ and is compost made by bean cake in AH.

“-” indicated there was no chemical/organic fertilizer input.
